# Interleukin-10 reorganizes the cytoskeleton of mature dendritic cells leading to their impaired biophysical properties and motilities

**DOI:** 10.1371/journal.pone.0172523

**Published:** 2017-02-24

**Authors:** Xiaoli Xu, Xianmei Liu, Jinhua Long, Zuquan Hu, Qinni Zheng, Chunlin Zhang, Long Li, Yun Wang, Yi Jia, Wei Qiu, Jing Zhou, Weijuan Yao, Zhu Zeng

**Affiliations:** 1 Key Laboratory of Biology and Medical Engineering, Guizhou Medical University, Guiyang, P.R. China; 2 Engineering Center of Medical Biotechnology Application, Guizhou Medical University, Guiyang, P.R. China; 3 School of Biology and Engineering, Guizhou Medical University, Guiyang, P.R. China; 4 Hemorheology Center, School of Basic Medical Sciences, Peking University Health Science Center, Beijing, P.R.China; 5 Department of Head and Neck, Affiliated Cancer Hospital, Guizhou Medical University, Guiyang, P.R.China; 6 Department of Nephropathy & Rheumatism, Third Affiliated Hospital, Guizhou Medical University, Duyun, P.R.China; George Mason University, UNITED STATES

## Abstract

Interlukin-10 (IL-10) is an immunomodulatory cytokine which predominantly induces immune-tolerance. It has been also identified as a major cytokine in the tumor microenvironment that markedly mediates tumor immune escape. Previous studies on the roles of IL-10 in tumor immunosuppression mainly focus on its biochemical effects. But the effects of IL-10 on the biophysical characteristics of immune cells are ill-defined. Dendritic cells (DCs) are the most potent antigen-presenting cells and play a key role in the anti-tumor immune response. IL-10 can affect the immune regulatory functions of DCs in various ways. In this study, we aim to explore the effects of IL-10 on the biophysical functions of mature DCs (mDCs). mDCs were treated with different concentrations of IL-10 and their biophysical characteristics were identified. The results showed that the biophysical properties of mDCs, including electrophoresis mobility, osmotic fragility and deformability, as well as their motilities, were impaired by IL-10. Meanwhile, the cytoskeleton (F-actin) of mDCs was reorganized by IL-10. IL-10 caused the alternations in the expressions of fasin1 and profilin1 as well as the phosphorylation of cofilin1 in a concentration-dependent fashion. Moreover, Fourier transformed infrared resonance data showed that IL-10 made the status of gene transcription and metabolic turnover of mDCs more active. These results demonstrate a new aspect of IL-10’s actions on the immune system and represent one of the mechanisms for immune escape of tumors. It may provide a valuable clue to optimize and improve the efficiency of DC-based immunotherapy against cancer.

## Introduction

Interleukin-10 (IL-10) is an immunomodulatory cytokine which is produced by a variety of cells, such as regulatory T lymphocytes subsets, monocytes, activated macrophages and other cells [1]. IL-10 predominantly induces immune tolerance and plays beneficial roles in some physiological and pathological processes such as pregnancy, tissue homeostasis and allergic diseases [2–5]. But in some cases, IL-10 is detrimental. It exists in the tumor microenvironments, especially for advanced tumors [6]. IL-10 mediates tumor immunosuppression and leads to the negative prognosis of tumor-bearing hosts [7–8], whose underlying mechanisms are that IL-10 could inhibit T cell proliferation and induce T cell to differentiate to the regulatory T cell phenotype [9]. IL-10 enhances the immune tolerance and weakens the effector T cell response to tumors through inhibiting the secretions of pro-inflammation factors, including IL-6, TNF-α, IL-12, INF-γ, etc.[1]. IL-10 also can down-regulate the expressions of MHC-II and co-stimulatory cytokines produced by antigen-presenting cells (APCs) such as dendritic cells (DCs) [10, 11] and impair APC’s T cell activation ability. Current evidences show that B cells expressing IL-10 could suppress the activities of cytotoxic CD4^+^ T cells in hepatocellular carcinoma, leading to the poor prognosis [12]. These studies indicate that IL-10 acts as a detrimental member in the tumor microenvironment. The recent studies on the roles of IL-10 in tumor immunosuppression mainly focus on its biochemical effects. However, the effects of IL-10 on the biophysical characteristics of immune cells are still elusive.

DCs are the most potent antigen-presenting cells and induce the differentiation of naïve T cells [13–15]. DC-based cancer vaccination is considered as one of the most promising therapies against cancer. It has been shown that DCs’ immune functions are impaired in tumor microenvironment [16]. Our previous work revealed that DCs at different differentiation stages possess distinct biophysical properties [17, 18]. More importantly, we found that the microenvironments of hepatocellular carcinoma and chronic myelogenous leukemia could greatly deteriorate the biophysical properties of DCs, such as deformation abilities and motilities, etc. [19, 20], indicating that the impaired biophysical properties of DCs could be one of reasons for tumor’s immune escape. Therefore, it is critical to better understand the mechanisms how tumor microenvironments affect the biophysical functions of DCs. The tumor microenvironment is a complex system, including various kinds of cells, e.g., tumor cells, immune cells, etc., and cytokines, e.g., IL-6, IL-10, TGF-β, etc. [21–24]. It would be useful to investigate the roles of each essential component in the regulation of DC functions. We have found that TGF-β_1_ had suppressive effects on DCs [25].

In the present study, we focused on the roles of IL-10 in the changes of DCs’ biophysical functions. Our results showed that the cytoskeleton (F-actin) of mDCs was reorganized by IL-10, resulting in their impaired biophysical characteristics and motilities, which were associated with the altered expression levels of some cytoskeleton-binding proteins. It's significant for further understanding the biological behaviors of DCs and immune escape mechanism of cancer, as well as how to enhance the efficiency of the DCs-based immunotherapy against cancer.

## Materials and methods

### Materials

Recombinant human granulocyte-macrophage colony-stimulating factor (rhGM-CSF), recombinant human interleukin-4 (rhIL-4), recombinant human tumor necrosis factor (rhTNF-α), interleukin-1β (IL-1β) and interleukin-10 (IL-10) were purchased from Peprotech Company (UK). FITC- or PE-conjugated mouse anti-human CD11c, CD40, CCR7, CD80, CD83, CD86 and HLA-DR antibodies were from Sigma (St. Louis, MO). Human CD14^+^ Monocytes Isolation Cocktail Kit was purchased from Miltenyi Company (Miltenyi Biotec, Bergisch Gladbach, Germany). Primary antibodies: anti-cofilin1, anti-phospholated cofilin1, anti-profilin1, anti-fascin1 and anti-β-actin antibodies were from Sigma. Human umbilical vein endothelial cells (HUVECs) were generously provided by Dr. Dai Xiaoqian from School of Public Health, Peking university Health Science Center. Fresh human peripheral venous blood of healthy volunteers with a same blood phenotype was obtained from Beijing Red Cross Blood Center. Written informed consent was obtained from all subjects for being included in the study. The whole study was approved by the ethics committees of Guizhou Medical University and Peking University Health Science Center.

### Dendritic cell culture

Dendritic cells were generated from human peripheral blood mononuclear cells (PBMCs) as described previously [18]. In brief, PBMCs were isolated from peripheral venous blood by Ficoll density gradient centrifugation. Highly enriched CD14^+^ monocytes were separated from PBMCs by using the cocktail immunomagnetic beads. Subsequently, the monocytes with 98% purity were cultured in RPMI 1640 complete medium (20% FBS, 1% glutamine, 1% penicillin/streptomycin, 1% Hepes) supplement with 150 ng/ml rhGM-CSF and 100 ng/ml rhIL-4 at 37°C in humidified 5% CO_2_. After 7 days, rhTNF-α (10 ng/ml) was added to culture media and the cells were further cultured for another 3 days to obtain mature DCs (mDCs). The expressions of surface markers including CD11c, CD40, CCR7, CD80, CD83, CD86 and HLA-DR were analyzed by flow cytometry.

### Treatment of mDCs with IL-10

mDCs were treated with IL-10 for 48h at 0, 0.01, 0.1, 1, 10 ng/ml, respectively, before further analyses were performed. The working concentrations and times of IL-10 were determined based on literatures [16, 26, 27] and our preliminary experiments.

### Analysis of cell apoptosis

IL-10 treated mDCs were stained with trypan blue and the cell viability was analyzed. The cells were also stained with Annexin V-FITC and propidium iodide (PI) and the cell apoptosis was analyzed by flow cytometry.

### Measurements of deformability, osmotic fragility, and electrophoretic mobility of IL-10-treated mDCs

The deformability, osmotic fragility and electrophoretic mobility (EPM) of IL-10-treated mDCs were measured following the previously described protocols [25]. Briefly, the deformability was measured with a micropipette system and defined as the ratios between the length of cell tongue aspirated into the micropipette and the radii of micropipettes. Osmotic fragility was measured by suspending the treated mDCs in hypotonic buffers with osmolality ranging from 25 to 295 mOsm/kg for 30 min. The numbers of non-hemolyzed cells were counted and the hemolysis rate was calculated. The EPMs of treated mDCs were measured with a cell electrophoresis meter (LIANG-100, Shanghai Medical University, China).

### Measurement of transendothelial migration of IL-10-treated mDCs by Transwell

HUVECs were seeded in the Transwell insert (5.0 μm pore) and grew for 48h to reach confluent. IL-10-treated mDCs were placed in the insert at a density of 1 ÿ 10^6^ cells/well. A chemoattractant, CCL19 (25 ng/ml), was added into the bottom compartment of the transwell. After the incubation of 12h at 37°C, the numbers of mDCs that have migrated into the bottom compartment were counted by a hematocytometer. The percentage of migrating cells was then calculated.

### F-actin organization and morphological changes in IL-10-treated mDCs

IL-10-treated mDCs were stained with rhodamine phalloidin (Invitrogen, USA) and their F-actin structures and morphologies were examined on a confocal laser scanning microscope (Leica, Germany). Images were acquired and the F-actin contents were quantified by measuring the mean fluorescent intensities in each group. The lengths and numbers of cell membrane protrusions were also measured using Leica LAS Image Analysis software [28]. At least twenty cells in each group were selected for analysis.

### Western blot assay

mDCs were harvested after treatment and lysed in RIPA buffer (20 mmol/L sodium phosphate, pH 7.4, 150 mmol/L sodium chloride, 1% Triton X-100, 5 mmol/L EDTA, 200 μmol/L phenymethylsulfonyl fluoride, 1 μg/ml aprotinin, 5 μg/ml leupeptin, 1 μg/ml pepstatin and 500 μmol/L Na_3_VO_4_). Total protein (50 μg) was separated on 12% SDS-PAGE gel and transferred onto a nitrocellulose membrane. After blocking, the membrane was incubated with anti-cofilin1, anti-phospholated cofilin1, anti-profilin1, anti-fascin1 and anti-β-actin antibodies, followed by horseradish peroxidase-conjugated goat anti mouse or rabbit IgG. The signals were visualized by chemiluminescent detection. The gray values of the signals were measured by Image J (NIH). The expression levels of proteins were normalized with β-actin.

### Fourier Transformed Infrared Resonance (FTIR)

2 × 10^6^/ml of IL-10-treated mDCs were washed with 0.9% NaCl and transferred onto the CaF_2_ crystal. The cells were dried at 37°C for 10min and a film of 2-3mm was formed. The CaF_2_ crystal was mounted in the sample holder and covered with another crystal. The infrared absorption spectrum was recorded using an Infrared Spectrometer (ENXUS-470 FT-IR). The scanning range was 400~4000cm^-1^ with the resolution of 8cm^-1^ and the scanning stack up to 256 times. The absorption spectrum of 0.9% NaCl was measured as the blank control. All the spectra were subtracted blank control. Fourier self-deconvolution was done with OMNIC6.0 software with broadband = 56.4 and sensitivity enhancement factor = 2.6 to get the deconvolution spectrum.

### Statistical analyses

All experiments were performed at least three times. The results were presented as mean ± standard deviation (SD). Analysis of variance (ANOVA two-way) of SPSS (11.5) was used for statistical data analyses. P<0.05 was considered statistical significance.

## Results

### IL-10 treatment did not affect the viability and apoptosis of mDCs

It has been shown that the biophysical characteristics of cells are closely correlated with apoptosis [29, 30]. We first examined the effect of IL-10 on the viability and apoptosis of mDCs. The results (Table 1) showed that the viabilities and apoptosis rates of mDCs were not affected by IL-10 treatment.

**Table 1:** Apoptosis and viability rates of mDCs cultured in different concentration IL-10 conditioned medium (Mean SD).

IL-10 (ng/ml)	0	0.01	0.1	1	10
Apoptosis rates (%)	4.21±1.22	3.69±1.53	3.47±1.48	3.72±1.33	4.18±1.17
Viability rates (%)	92.31±4.22	89.67±3.59	88.39±2.47	85.66±3.35	87.29±3.56

### IL-10 deteriorated the biophysical properties of mDCs

**Fig 1:**
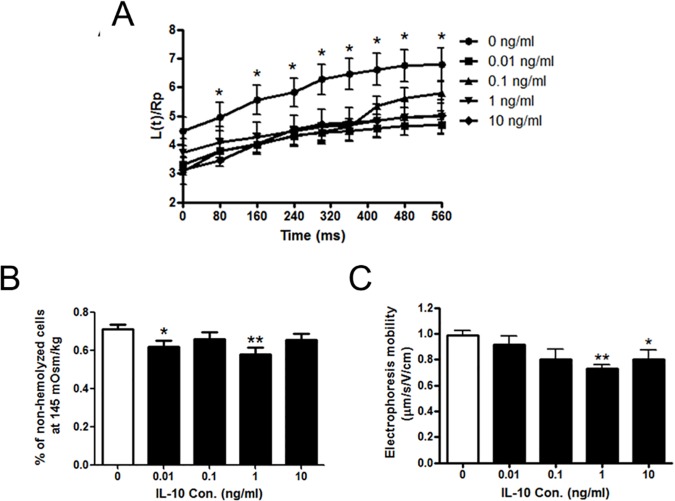
The effects of IL-10 on the biophysical properties of mDCs. (A) The deformation of mDCs treated by IL-10 measured by micropipette aspiration. The ratios between the length of cell tongue aspirated into the micropipette, L(t), and the radius of pipette, Rp, were plotted against the time of aspiration. (B) The percentage of non-hemolyzed cells at osmotic pressure of 145mOsm/kg. (C) Electrophoretic mobilities of mDCs treated by IL-10. Compared with control group: *p<0.05, **p<0.01.

We next investigated the effects of IL-10 on the biophysical characteristics of mDCs, including the deformability, osmotic fragility and electrophoresis mobility. Micropipette aspiration system was employed to measure the deformability of IL-10-treated mDCs. Data (Fig 1A) showed that the ratios of L(t)/Rp were much less in different groups of IL-10-treated mDCs than un-treated mDCs (p<0.05), indicating that IL-10 made mDCs much stiffer and less deformable. Then IL-10-treated mDCs were subjected to hypotonic solutions for osmotic fragility test. Data (Fig 1B) showed that, at 145mOsm/kg osmotic pressure, the percentages of non-hemolyzed cells were significantly lower in 0.01 and 1 ng/ml IL-10-treated mDCs (p<0.05 and p<0.01, respectively) in comparison with those of untreated cells, suggesting that mDCs could not bear the hypotonic stress and have higher osmotic fragility after IL-10 treatment. We also measured the electrophoretic mobility (EPM) to assess the density of surface charge on IL-10-treated mDCs. We found that the EPM decreased after IL-10 treatment and reached the lowest levels at 1 and 10 ng/ml (Fig 1C, p<0.01 and p<0.05, respectively), suggesting that the surface charge was reduced by IL-10. These findings indicated that IL-10 changed the biophysical properties of mDCs.

### IL-10 reduced the transendothelial migration of mDCs

**Fig 2:**
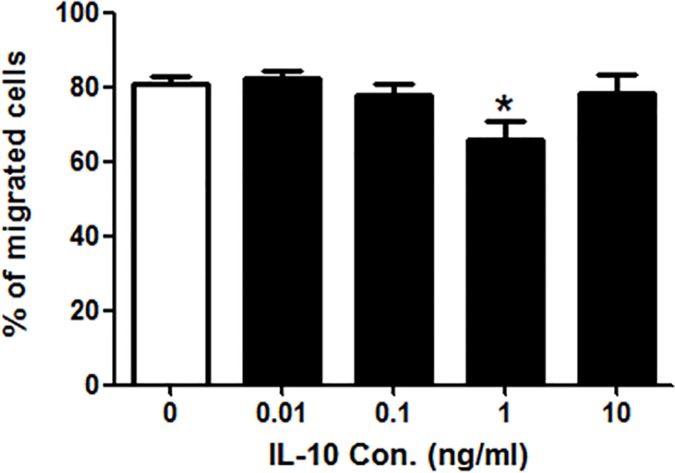
The effect of IL-10 on the transendothelial migration (TM) of mDCs. The migrations of IL-10-treated mDCs were determined by Transwell assay. The migrated cells were counted using a haemmacytometer. Compared with control group: *p<0.05.

The transendothelial migration is an important capability of DCs which is gradually enhanced during their differentiation. The impaired biophysical properties may result in the change of migration. A transwell assay was performed to test the transendothelial migration capability of IL-10-treated mDCs. Data showed that, after the treatment of 1 ng/ml IL-10, the number of migrated mDCs was much less than those of untreated mDCs (p <0.05), while there was no change in other groups (Fig 2), this suggesting that IL-10 could reduce the transendothelial migration of mDCs at 1 ng/ml.

### IL-10 reorganized the F-actin structure in mDCs

**Fig 3:**
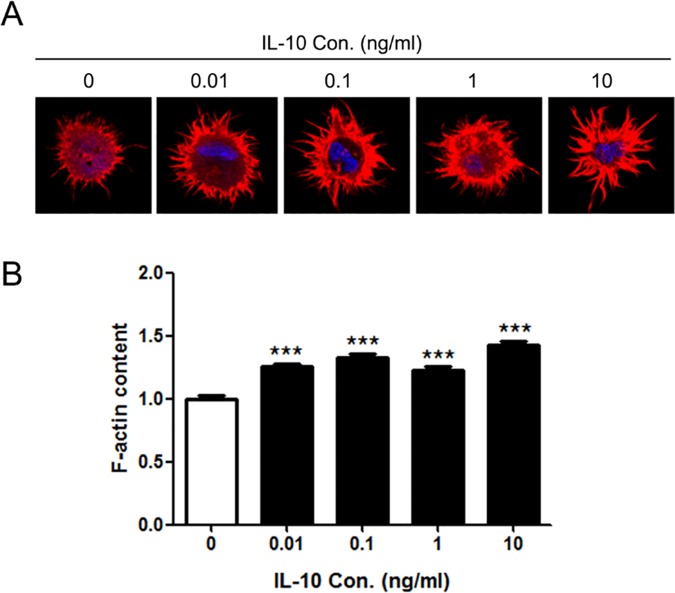
The effect of IL-10 on the F-actin content of mDCs. (A) The F-actin organization of mDCs treated by different concentrations of IL-10. Cells were labeled with rhodmine phalloidin and photographed by a confocal microscope (600×). (B) F-actin contents of IL-10-treated mDCs. F-actin contents were quantified by measuring the mean fluorescent intensities of F-actin. Compared with control group: ***p<0.001.

The deformability and migration of cells are closely associated with the organizations of cytoskeleton, especially the organizations of filamentous actin (F-actin). Our previous studies have shown that the F-actin cytoskeleton of DCs dramatically reorganized in the tumor microenvironments of hepatic carcinoma and leukemia [19, 20]. Therefore, we analyzed whether IL-10 would affect the F-actin of mDCs. Confocal microscopy analysis (Fig 3) showed that, after treated by different concentrations of IL-10, the F-actin structures of mDCs were markedly reorganized and F-actin contents in cells were enhanced as compared with those of control (p<0.001). In IL-10-treated mDCs, F-actin was accumulated in the protrusions while there was much less F-actin in the cytoplasm. It is interesting to notice that the lengths of protrusions appeared much longer in IL-10-treated mDCs than those of untreated mDCs. These data and observations suggest that IL-10 caused the changes in both content and organization of F-actin in mDCs.

### IL-10 caused the alterations in the expression levels of fascin1 and profilin1 as well as phosphorylation of cofilin1

**Fig 4:**
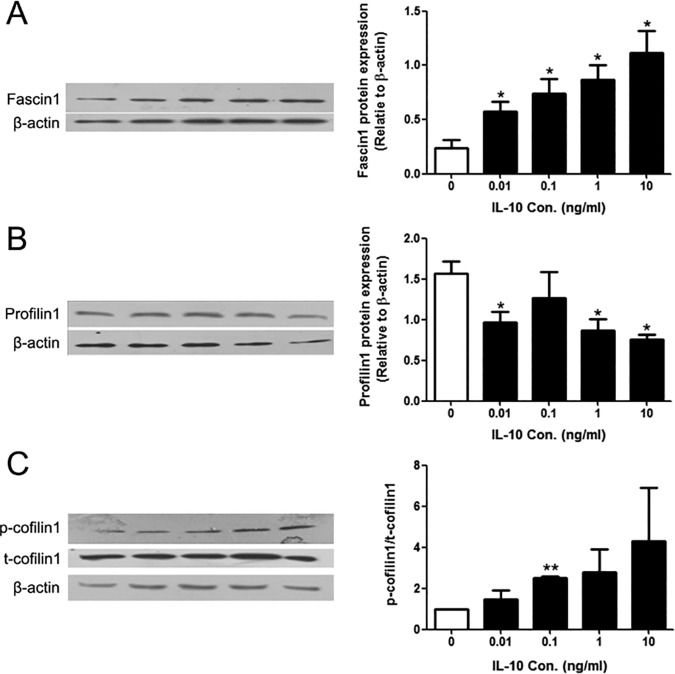
The effects of IL-10 on the expression levels of some actin-binging proteins in mDCs. (A) Representative Western blot image of fascin1 (left panel) and the quantitative data (right panel). (B) Representative Western blot image of profilin1 (left panel) and the quantitative data (right panel). (C) Representative Western blot image of p-cofilin1, total cofilin1 (left panel) and the quantitative data (right panel). β-actin was used as the internal control. Compared with control group: *p < 0.05, **p<0.01.

F-actin is the highly dynamic structure which is regulated by multiple actin-binging proteins. Precise spatio-temporal expression of the actin-binging proteins ensures the proper cytoskeleton organization and normal cell functions. Since F-actin content and transendothelial migration were altered by IL-10 treatment, we examined the expression levels and activation of some actin-binging proteins, such as fascin1, profilin1, and cofilin1. Western blotting data showed that IL-10 treatment caused the alterations in all three proteins in a concentration-dependent manner (Fig 4A–4C): fascin1 expression was greatly increased (p<0.05), while profilin1 expression was reduced (p<0.05); the ratios of phosphorylated cofilin1 (p-cofilin1) to total cofilin1 (t-cofilin1) were increased (p<0.01).

### IL-10 caused the changes in the Fourier Transform Infrared spectrum (FTIR) of mDCs

The peak values of the FTIR spectra at different wave numbers are related to the various cell components, such as proteins, membrane lipids, and nucleic acids [16]. Several groups showed that the ratios of absorption intensity of A_1020_/A_1545_, A_1121_/A_1545_, A_1030_/A_1080_ and A_1030_/A_2924_ represent DNA/amideII (relative contents of DNA and proteins), RNA/amideII (gene transcriptional status), glucose/phosphate (metabolic turnover) and glucose/phospholipids (*de novo* synthesis of phospholipids at the expense of free glucose), respectively [18, 31, 32]. As shown in Table 2, the ratios of A_1020_/A_1545_, A_1121_/A_1545_ and A_1030_/A_1080_ significantly increased in mDCs treated with 0.01 ng/ml IL-10. The ratio of A_1121_/A_1545_ also enhanced in 0.1 ng/ml IL-10-treated mDCs (p<0.05). But the ratios in other groups were not different to those of control. These data suggested that the treatment of 0.01 and 0.1ng/ml of IL-10 made the status of gene transcription and metabolic turnover of mDCs more active.

**Table 2:** Infrared absorption intensities of mDCs cultured in different concentration IL-10 conditioned medium (Mean SD).

IL-10 (ng/ml)	0	0.01	0.1	1	10
A_1020_/A_1545_	0.323±0.532	1.788±1.098*	0.363±0.325	0.567±0.751	0.341±0.452
A_1121_/A_1545_	0.125±0.113	0.732±0.609*	0.383±0.218*	0.532±0.700	0.278±0.358
A_1030_/A_1080_	0.411±0.622	3.598±2.870*	1.832±2.053	2.298±4.053	0.814±1.287
A_1030_/A_2924_	2.335±3.770	1.381±0.045	0.642±0.281	0.449±0.250	0.645±0.238

Compared with control

^*^P<0.05.

## Discussion

DCs has been utilized as the tumor vaccination and applied to clinic [33–35]. But the efficiency of the DC-based immunotherapy against cancer is unsatisfactory. IL-10 is one of the main tumor-suppression factors which produced by most of cancers. It contributes to immune dysfunction in tumor microenvironment through damping the functions of immune cells, including T cells, B cells, macrophages, NK cells and DCs [36–38]. It has been shown that circulating concentrations of IL-10 were raised in 13 different cancer types and were associated with adverse disease stage or with negative prognosis [26]. In healthy people, the serum IL-10 level is at the range of 3.31±0.42 pg/ml [39]. But it is about 13.69±1.80 pg/ml in patients with hepatocellular carcinoma [39] and 154.55±45.31 pg/ml in patients with breast cancer [40]. Researchers also found that several human multiple myeloma cell lines could spontaneously produce IL-10 up to 179 pg/ml and their IL-10 production could raised up to 1626 pg/ml when they were stimulated by IL-6 [41]. In the present studies, we used different concentrations of IL-10 ranging from 0.01–10 ng/ml and examined its influences on mDCs’ biophysical characteristics.

EPM is one of the biophysical parameters, which reflect the negative charges on the surface of cell membrane. Our previous studies have shown that DCs’ EPM continuously increased during their differentiation process [42]. Here, we found that EPMs of mDCs were decreased after the treatment of IL-10 (Fig 1), suggesting that the surface charges on the membrane of mDCs were reduced. It could be inferred that the reducing of the surface charges would decrease the repulsion forces between mDCs and other cells and/or extracellular matrix, enhancing their adhesion and interaction leading to the affected ongoing immune response. Previous studies revealed that antigen-carrying mDCs need low adhesion ability and high migration velocity in order to arrive at the secondary lymphoid tissue to perform their antigen-presenting functions [17, 43]. The adhesion is irrelevant to mDCs’ migration since their adhesion ability is dramatically reduced as compared to immature DCs (imDCs) [44]. Some researchers also found that antigen loaded mDCs use an adhesion-independent migration strategy from the periphery to the draining lymph nodes [45]. Our results showed that the transendothelial migration capacity of mDCs was reduced by 1 ng/ml IL-10 (Fig 2). So it is reasonable to speculate that IL-10 may impair the migration ability of mDCs through enhancing their adhesion with other cells or tissue.

Meanwhile, our results showed that the deformability of mDCs was deteriorated by IL-10 (Fig 1A). The higher osmotic fragilities also indicate their deteriorated deformability (Fig 1B). Better deformability is helpful for migration when the cells squeeze through the blood vessel, lymphatic vessel, the junctions between cells and complex extracellular matrix. Our previous studies found that mDCs have the highest deformability at DCs’ different differentiation stages from monocytes [18], suggesting that cell deformability is essential for mDCs during their migration. The defective deformability of mDCs might also contribute to a flawed migration. These data indicated that IL-10 could deteriorate the electronic characteristic and deformability of mDCs and weaken their migration ability. This might be one aspect of the cancer immune escape. It also explained why very low percentage (<1%) of *ex vivo* DCs could reach the lymph nodes after their intracutaneous injection into a host loading tumor [46, 47].

Our previous studies revealed that DCs had changes in the osmotic fragility at different differentiation stages [18]. Compared with imDCs, mDCs have a higher osmotic fragility and it can be further elevated in the tumor microenvironment [18]. Here, we found that IL-10 treatment increased the mDCs’ osmotic fragilities (Fig 1B). This would suggest that antigen-loading mDCs may not be able to resist the environmental changes in the tumor-bearing hosts. This could be another reason for the immune escape of tumors. We noticed that, in the dose responses of IL-10 in osmotic fragility, EPM and transendothelial migration, it seemed to have an interesting peak at 1 ng/ml and increasing the concentration to 10 ng/ml resulted in a loss of response (Figs 1B, 1C and 2). This is reasonable because the serum concentrations of IL-10 in cancer patients and the secretion of IL-10 by cancer cell lines are in the range of 0.01, 0.1, and 1 ng/ml [39–41]. The effects of IL-10 on the biophysical properties of DCs might be very efficient in this range.

Dynamic organization of actin cytoskeleton is essential to DCs’ biological functions, including antigen capture, amoeboid migration, deformability, and forming immunological synapse [48–50]. In this study, we found that F-actin contents of mDCs were significantly elevated by IL-10 and large amount of F-actin were concentrated in the cell peripheral and protrusions (Fig 3). Fallquvist et al. found that depolymerization of F-actin could reduce the cell stiffness and increase the relaxation behavior of fibroblasts [51]. So it is reasonable to speculate that the increased F-actin content would elevate the stiffness of mDCs, thus leading to their poor deformabilities.

It’s well known that a variety of actin-binging proteins modulates actin cytoskeletal organization [52]. In our experiments, some proteins including fascin1, profilin1, and confilin1 were selected to investigate, which participate in the regulations of actin cytoskeleton and cell migration [53–55]. We found that IL-10 could affect the expressions of fascin1 and profilin1 as well as the phosphorylation of cofilin1 in a concentration-dependent fashion (Fig 4).

Confilin1 belongs to actin-depolymerizing factor (ADF)/cofilin family proteins that drive depolymerization of F-actin [52], including non-phosphorylation and phosphorylation states [56, 57]. The former is the active phenotype which promotes separation of ADP-F-actin from the pointed end of F-actin, while the latter is the inactive phenotype which maintains polymerization and stabilization of F-actin and suppresses actin turnover [58]. The balance between p-cofilin1 and cofilin1 plays a key role in maintaining the F-actin dynamics. Increasing cofilin1 phosphorylation could decrease depolymerization of F-actin [57]. Thus, the elevated expression lever of p-cofilin1 in mDCs after treated by IL-10 might lead to a higher content of F-actin and longer protrusions. In addition, the dynamical equilibrium between p-cofilin1 and cofilin1 closely associated with cell migration [53]. The elevated cofilin1 phosphorylation could lead to an aberrant accumulation of F-actin in the leading edge of the mobile cells and restrain the cell motility [53]. Our results are in consistent with these findings.

Profilin1 is another important protein with a major function of regulating actin organization. Moreover, profilin1 has an indispensable and complex role in cell migration: positive regulation in normal cells through facilitating the formation of membrane protrusion and maintaining their dynamics during the cell migration such as vascular endothelial cell (VECs) [59], while negative regulation in adenocarcinoma cells and normal human mammary epithelial cells (HMECs) through a contrary way [60]. Our data showed that the expression levels of profilin1 were decreased in IL-10-treated mDCs, in accordance with their increased F-actin content and decreased migration ability (Figs 2–4). Fascin1 is an actin-bundling protein which is highly expressed in mDCs [61]. Studies have demonstrated that fascin1 plays a crucial role on filopadia assembly and dendrite formation [62, 63]. In the present study, after treatment by IL-10, the expression levels of fascin1 in mDCs were up-regulated, which would be related to the elevation of their membrane protrusion. Fascin1 can depolymerize podosome, a special structure in imDCs for cell-matrix adhesion, and increase dynamics of membrane protrusions and then positively regulate migration of mDCs into lymph nodes [64]. But this was in contradiction with our result that the treated-mDCs had deteriorated motilities. It could be reckoned that other cytoskeleton-binding proteins might take part in this process, which are needed to further investigate in detail. Moreover, we found that the phosphorylation of signal transducer and activator of transcription 3 (STAT3), a critical molecule in IL-10 signaling pathway, increased in IL-10-treated mDCs (data not shown). The activation of STAT3 might be responsible for the changes occurred in the actin-binding proteins we detected since the gene expressions of fascin1 and profilin1 are regulated in a STAT3-dependent manner [65, 66].

The biophysical characteristics of cell can be changed by its apoptosis or viability [30]. Our data (Table 1) showed that IL-10 did not induce mDCs’ apoptosis, suggesting that the changed biophysical characteristics of mDCs did not attribute to cell apoptosis. Our group and other researchers found that FTIR spectra technology can be applied to investigate the functional status of cells [67]. The ratios between the absorptions at certain wave numbers represent the proliferation, gene transcription state and metabolic turnover, and usage of free glucose [31, 32]. Our data showed that the treatment of 0.01 and 0.1ng/ml of IL-10 made the status of gene transcription and metabolic turnover of mDCs more active, suggesting that mDCs could be at a stress status in response to IL-10 treatment. This seems contradictory to our previous finding showing that the gene transcription activity and energy states of mDCs are specifically suppressed by hepatocellular carcinoma cell line (HCC) Bel7402 [68]. Since HCC cells secrete many kinds of cytokines including IL-10, VEGF, TGF-β1, etc., our previous data are the overall effect of HCC-derived all cytokines, which may be different from the effect of one specific cytokine, IL-10. We have performed microarray analyses on IL-10-treated mDCs [69]. The gene ontology (GO) and Kyoto Encyclopedia of Genes and Genomes (KEGG) analyses on the up-regulated genes showed that processes of RNA polymerase II regulation and DNA-dependent transcription regulation and some metabolism pathways, such as tricarboxylic acid cycle (TCA cycle), glutamine metabolism and pyruvate metabolism, etc., were all increased by IL-10 treatment. These were in accordance with FTIR data.

## Conclusion

In summary, the present study indicated that IL-10 modulated immunosuppression not only biochemically but also biophysically. This is the first time to show that IL-10 could disturb the actin cytoskeleton of mDCs and deteriorate their biophysical features and motilities, which might be a new aspect of IL-10’s actions on the immune system and represents one of aspects of tumor immune escape. These findings may provide valuable clues to optimize and improve the efficiency of DCs-based immunotherapy against cancer.
